# Emotion Recognition from rPPG via Physiologically Inspired Temporal Encoding and Attention-Based Curriculum Learning

**DOI:** 10.3390/s25133995

**Published:** 2025-06-26

**Authors:** Changmin Lee, Hyunwoo Lee, Mincheol Whang

**Affiliations:** 1 Department of Human-Centered Artificial Intelligence, Sangmyung University, Seoul 03016, Republic of Korea; ckdals1380@gmail.com; 2 Department of Emotion Engineering, Sangmyung University, Seoul 03016, Republic of Korea; lhw4846@naver.com

**Keywords:** remote photoplethysmography, affective computing, temporal dynamics, sparse attention, emotion recognition, curriculum learning, autonomic nervous system, physiological computing

## Abstract

**Highlights:**

**What are the main findings?**

- A temporal-only rPPG framework with a multi-scale CNN, sparse α-Entmax attention, and Gated Pooling achieved 66.04% accuracy and a 61.97% weighted F1-score for arousal on MAHNOB-HCI (mixed subjects).
- The model underperformed for valence (62.26% accuracy), highlighting the physiological limits of unimodal time-series signals.

**What are the implications of the main findings?**

- Temporal rPPG can rival other single-modality methods for arousal when physiologically inspired temporal modeling is applied.
- Addressing valence requires the integration of spatial or multimodal cues, guiding future affective computing designs.

**Abstract:**

Remote photoplethysmography (rPPG) enables non-contact physiological measurement for emotion recognition, yet the temporally sparse nature of emotional cardiovascular responses, intrinsic measurement noise, weak session-level labels, and subtle correlates of valence pose critical challenges. To address these issues, we propose a physiologically inspired deep learning framework comprising a Multi-scale Temporal Dynamics Encoder (MTDE) to capture autonomic nervous system dynamics across multiple timescales, an adaptive sparse α-Entmax attention mechanism to identify salient emotional segments amidst noisy signals, Gated Temporal Pooling for the robust aggregation of emotional features, and a structured three-phase curriculum learning strategy to systematically handle temporal sparsity, weak labels, and noise. Evaluated on the MAHNOB-HCI dataset (27 subjects and 527 sessions with a subject-mixed split), our temporal-only model achieved competitive performance in arousal recognition (66.04% accuracy; 61.97% weighted F1-score), surpassing prior CNN-LSTM baselines. However, lower performance in valence (62.26% accuracy) revealed inherent physiological limitations regarding a unimodal temporal cardiovascular analysis. These findings establish clear benchmarks for temporal-only rPPG emotion recognition and underscore the necessity of incorporating spatial or multimodal information to effectively capture nuanced emotional dimensions such as valence, guiding future research directions in affective computing.

## 1. Introduction

Emotion recognition is a fundamental component of affective computing and human–computer interaction, with significant implications across healthcare, education, and consumer technologies [1,2]. Traditional methods primarily rely on observable cues such as facial expressions or speech. However, these external indicators can be intentionally controlled or masked, limiting their reliability in representing genuine emotional states [3]. Physiological signals—such as heart rate, blood volume pulse (BVP), and skin conductance—regulated by the autonomic nervous system (ANS), offer a more authentic and less voluntarily modifiable reflection of emotional states, making them ideal for unobtrusive affective computing [4].

Extensive psychophysiological research has firmly established that emotional states trigger characteristic, transient changes in cardiovascular activity [4]. Heart rate variability (HRV), defined by fluctuations in intervals between heartbeats, reflects ANS activity, distinctly correlating with emotional regulation processes [5]. Additionally, pulse waveform morphology captures vascular tone variations associated directly with emotional arousal [6]. Notably, these physiological responses are transient, sparsely distributed, and exhibit non-uniform temporal patterns [4,7]—highlighting a critical gap in current approaches: effective identification and the interpretation of emotionally salient temporal segments amidst noisy physiological signals.

Recent advancements in remote photoplethysmography (rPPG) enable unobtrusive, camera-based monitoring of cardiovascular activity by measuring subtle skin color variations induced by cardiac pulse waves [8]. This technique allows scalable affective computing applications across diverse industrial contexts due to the proliferation of camera-equipped devices (e.g., smartphones, laptops, and surveillance systems), significantly broadening the practical utility of emotion recognition technology [9].

Despite promising potential, recognizing emotions exclusively from unimodal temporal rPPG signals has significant unresolved challenges. Firstly, emotional physiological responses often manifest briefly and sporadically rather than continuously, complicating effective temporal analysis [4]. Secondly, rPPG signals inherently suffer from noise and artifacts from subject motion and environmental lighting changes compared to contact-based methods, impairing robust interpretation of subtle emotional cues [8,9]. Thirdly, typical session-level annotations induce a weak-label Multiple Instance Learning (MIL) scenario [10], necessitating sophisticated models to pinpoint informative temporal segments accurately, a challenge recently tackled in rPPG with domain adaptation techniques [11]. Fourthly, recognizing valence (the pleasantness of an emotion) from physiological signals remains inherently more challenging than arousal (the intensity of an emotion) as its correlates are more complex and less directly tied to general ANS activation [4,12]. Furthermore, large-scale, deep-learning-ready rPPG corpora remain rare; most public datasets contain <50 participants and fewer than 10 sessions per subject, thereby limiting the statistical power of data-hungry models such as LSTM or Transformer variants [13,14].

Consequently, we deliberately restrict our first evaluation to the well-controlled MAHNOB-HCI benchmark—which provides a relatively large number of sessions per subject—to isolate the intrinsic capabilities and limits of temporal-only rPPG before tackling cross-dataset generalization in future work in Section 5.2.

Our study explicitly addresses these challenges by proposing a novel deep learning framework. By processing signals in short, localized temporal chunks, we effectively isolate and analyze transient physiological responses. We introduce a Multi-scale Temporal Dynamics Encoder (MTDE), physiologically motivated by multi-rate ANS response characteristics [5,15] and designed to capture subtle temporal patterns across different timescales [16]. Furthermore, an adaptive sparse attention mechanism leveraging α-Entmax and entropy regularization explicitly identifies and prioritizes temporally sparse emotional segments, emulating selective human attentional processes [17,18,19]. A novel Gated Temporal Pooling mechanism robustly aggregates chunk-level information, effectively filtering noise through joint temporal weighting and feature-level gating [20].

Critically, we employ a physiologically inspired, three-phase curriculum learning strategy—exploration, discrimination, and exploitation—mirroring human attentional refinement and developmental learning principles [21,22]. This systematic training approach addresses weak-label issues, temporal sparsity, and signal noise incrementally, enabling stable, progressive learning from complex, noisy temporal data. To robustly evaluate our method, we employ weighted F1-scores, addressing class imbalance more objectively compared to prior work such as that by Mellouk & Handouzi [23]. This work therefore establishes a transparent baseline that future research in cross-dataset generalization and domain adaptation can build upon. Our evaluations on the MAHNOB-HCI dataset show promising results: for arousal, it achieves an accuracy of 66%, an F1-score of 0.7429, and a weighted F1-score of 0.6224; for valence, it achieves an accuracy of 62.3%, an F1-score of 0.6667, and a weighted F1-score of 0.6227, underscoring our model’s effectiveness despite inherent challenges.

Our contributions explicitly bridge critical research gaps, providing clear advancements:

- Focused Temporal Analysis of rPPG: This method establishes foundational insights into the capabilities and limitations of using exclusively temporal physiological information, providing a rigorous benchmark.
- Multi-scale Temporal Dynamics Encoder (MTDE): This effectively captures physiologically meaningful ANS responses across multiple timescales, addressing complexity in subtle temporal emotional signals.
- Adaptive Sparse Attention: This method precisely identifies transient, emotionally relevant physiological segments amidst noisy rPPG data, significantly enhancing robustness.
- Gated Temporal Pooling: This method sophisticatedly aggregates emotional information across temporal chunks, effectively mitigating noise and irrelevant features.
- Curriculum Learning Strategy: This method systematically addresses learning complexities associated with weak labels, noise, and temporal sparsity, ensuring robust, stable model learning.

## 2. Related Work

### 2.1. The Physiological Signals for Emotion Recognition

Cardiovascular activity is tightly coupled with autonomic nervous system (ANS) dynamics and therefore provides a robust, involuntary window on affective states [4,24]. Two families of descriptors are especially relevant:

- Heart-rate variability (HRV). Spectral power in the very-low-, low- and high-frequency bands maps onto distinct sympathetic–parasympathetic rhythms and has been linked to both arousal and valence [5,15].
- Pulse-wave morphology. Beat-to-beat amplitude and upslope changes correlate with rapid sympathetic activation, while longer-term contour variations reflect vagal tone [25,26].

Early affective computing systems relied on hand-crafted HRV indices (e.g., LF/HF ratio and SDNN). Later work introduced shallow CNNs or temporal convolutional networks (TCNs) that learn nonlinear dynamics directly from the waveform [16].

Our Multi-scale Temporal Dynamics Encoder (MTDE) builds upon this principle by operationalizing physiological insights into its architecture. Its three one-dimensional convolutional branches have effective receptive fields (≈0.2 s, 2 s, and 4 s) that are designed to intentionally cover the canonical HF, LF, and VLF HRV windows [5,15]. As validated by a kernel-size sensitivity study in Section 4.2, this physiologically aligned design yields superior performance compared to single-scale or randomly dilated variants.

### 2.2. Remote PPG Signal Extraction and Denoising

Remote PPG (rPPG) estimates the blood volume pulse from skin-color variations in ordinary video but is highly sensitive to illumination changes, motion, and camera noise [9,27]. Public rPPG–emotion databases are also small (<50 subjects) and heterogeneous in frame rate or lighting, limiting large-scale learning [9,28].

Recent end-to-end extractors—PhysNet [29], PhysFormer [30], RhythmFormer [31], and PhysMamba [32]—combine long-range sequence modeling with explicit noise suppression. We adopt PhysMamba [32] as a front-end because it (i) achieves the highest signal-to-noise ratio on MAHNOB-HCI, (ii) is open-source, and (iii) provides a stable, high-quality BVP signal. This approach allows for a focused analysis of the downstream emotion recognition stage as the influence of signal extraction quality is minimized.

### 2.3. Emotion Recognition from rPPG/PPG

Most previous rPPG affect studies fuse the pulse trace with facial appearance cues, obscuring the contribution of the temporal signal itself [9]. The few unimodal–temporal approaches can be grouped as follows:

- CNN-LSTM. Mellouk & Handouzi [23] combine 2D convolutions with an LSTM on four-second segments and report the first subject-balanced results on MAHNOB-HCI. The network architecture and training protocol are fully specified, making it the most appropriate domain-equivalent baseline.
- TCN and Transformer variants. Later works explored purely convolutional temporal blocks [16] or self-attention for longer contexts [30,31], but most studies used proprietary datasets or combined spatial features, preventing a direct apples-to-apples comparison on rPPG alone.

Given this landscape, CNN-LSTM serves as our primary, temporally focused reference. To further contextualize our model’s performance against non-recurrent architectures, additional various backbones including lightweight eight-layer TCNs are implemented as a comparison baseline (see Section 4.2 and Appendix E).

### 2.4. Comparison and Key Differences

**Figure 5:**
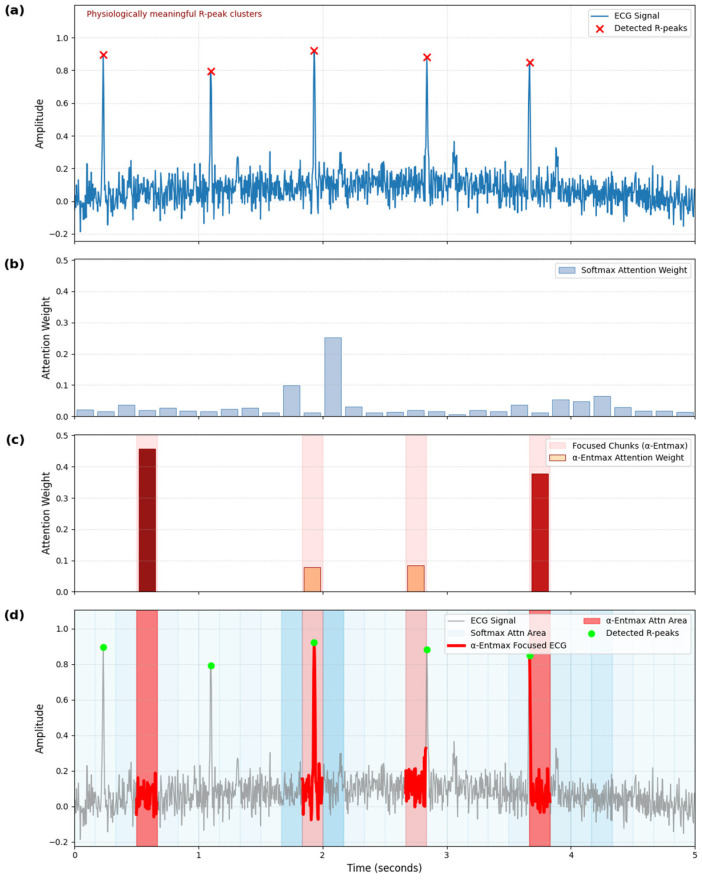
Qualitative analysis of sparse attention mechanism on ECG signal. This figure presents a comparative qualitative analysis of the attention distributions from a standard SoftMax model and our proposed α-Entmax sparse attention model on a representative ECG signal from a random subject’s random session. (**a**) displays the ECG signal with physiologically meaningful R-peaks highlighted. (**b**) shows the diffuse attention distribution of SoftMax (Entropy: 2.92). (**c**) illustrates the highly sparse attention distribution of α-Entmax (Entropy: 1.14). (**d**) overlays both attention distributions on the ECG signal, demonstrating how α-Entmax selectively focuses on specific physiological events, particularly R-peak clusters.

In summary, our work is distinguished from prior studies in several key aspects. The following points highlight our main contributions:

- Temporal-only perspective. We analyze the BVP waveform in isolation, establishing a lower-bound benchmark that future multimodal extensions can build upon.
- Physiology-guided multi-scale encoding. MTDE hard-codes HRV-aligned receptive fields [22,30] and is empirically validated via an ablation on kernel size.
- Interpretable sparse attention. α-Entmax [16] learns a few high-confidence temporal “spikes”; heat-map visualizations (Figure 5) coincide with sympathetic bursts in the waveform, aligning with theories of selective attention [19].
- Noise-robust aggregation. Gated Pooling jointly weights time steps and feature channels, reducing artifacts propagated from the rPPG extractor.
- Rigorous protocol and efficiency. A dedicated hold-out test set, weighted F1 scoring, and a 198 k-parameter model that runs at 0.66 s per two-minute video establish (Section 3.7) both statistical and practical credibility.

Together, these contributions define the first systematically evaluated baseline for temporal-only rPPG emotion recognition and lay the groundwork for future cross-dataset or domain adaptation studies.

## 3. Methodology

This section delineates the methodology employed in our study for emotion recognition from remote photoplethysmography (rPPG) signals. We describe the dataset utilized, the preprocessing steps, the overall framework architecture, the detailed components of our model, the physiologically inspired curriculum learning strategy, the evaluation metrics and comparative baseline, and the experimental setup.

### 3.1. Dataset and Experimental Protocol

#### 3.1.1. MAHNOB-HCI Dataset

We employ the publicly available MAHNOB-HCI multimodal emotion dataset, which comprises recordings from 27 subjects who viewed 20 emotional film clips [28]. The dataset represents one of the most comprehensive multimodal emotion databases with synchronized physiological signals, facial videos, and subjective ratings. After excluding unusable data from 3 subjects, a total of 527 valid sessions were utilized for this study. The dataset provides synchronized facial videos and ground-truth physiological signals, including electrocardiogram (ECG) data recorded at a 256 Hz sampling rate.

All videos were downsampled to 30 fps, a rate validated as sufficient for capturing subtle cardiovascular dynamics and maintaining HRV analysis accuracy. Studies have demonstrated that frame rates of 30 fps or higher preserve the temporal resolution necessary for accurate heart rate variability measurements. For classification, the self-reported valence and arousal ratings (on a 1–9 scale) were binarized into “low” (ratings 1–4) and “high” (ratings 5–9) classes by thresholding at the midpoint (4.5), following established protocols in affective computing research [33].

#### 3.1.2. Data Partitioning and Protocol Rationale

To maximize the data available for our deep learning model, the 527 sessions were partitioned into training (80%), validation (10%), and testing (10%) sets using a session-based random split. We acknowledge that a strict subject-independent split is the gold standard for evaluating generalization. However, as established in systematic reviews, the landscape of public cardiovascular-based emotion datasets is characterized by limited scale; a comprehensive survey of 18 such datasets revealed that most include fewer than 50 participants, with many offering limited session diversity per subject [6]. This data scarcity poses a significant challenge for training data-hungry deep learning models.

Consequently, we opted for this subject-mixed protocol to facilitate a thorough investigation of our proposed architecture’s capabilities on a rich set of session data. We explicitly acknowledge that this protocol may lead to an overestimation of the model’s generalization capability to entirely unseen subjects, a limitation that, along with the challenges of cross-dataset validation, is further addressed in our discussion (Section 5.1).

#### 3.1.3. A Two-Stage Process for Optimal Method Selection

To ensure a high-quality physiological input, we conducted a rigorous, two-stage experiment to select the optimal rPPG method. The MAHNOB-HCI dataset provides ground-truth physiological signals, including ECG data; however, it does not include raw contact-based photoplethysmography (PPG) signals for direct comparison. As a ground-truth heart rate (HR), we processed the dataset’s raw ECG signals; R-peaks were detected using the Pan–Tompkins algorithm [34] with a 0.5–40 Hz band-pass filter, and the resulting inter-beat intervals (IBIs) were used to calculate HR (HR = 60/IBI), which was then resampled to 30 Hz.

First, a broad comparison of traditional unsupervised algorithms against state-of-the-art deep learning models was conducted on our target dataset. The accuracy of the extracted signals was measured against the ECG-based ground-truth using Mean Absolute Error (MAE) in beats per minute (bpm) and Root Mean Square Error (RMSE). As summarized in Table 1, deep-learning-based methods demonstrated vastly superior accuracy, with PhysFormer [30] and PhysMamba [32] (both pre-trained on UBFC-rPPG) emerging as the top two performers.

Second, to make a final selection, we conducted a head-to-head analysis focusing on signal quality (SNR) and computational efficiency (VRAM usage). This test was performed using the standard UBFC-rPPG intra-dataset protocol [35]. The results in Table 2 clearly favor PhysMamba, which achieved a significantly higher SNR (3.67 vs. 0.25) and lower VRAM usage.

#### 3.1.4. Final Method and Implementation

While our validation on MAHNOB-HCI yielded an MAE of 5.38 bpm for HR estimation, our primary goal is to obtain a high-fidelity BVP waveform for learning subtle affective features. Research has demonstrated that the morphological characteristics of PPG signals contain essential physiological information for emotion recognition, with signal quality being more critical than absolute HR accuracy for the effective classification of affective state [6,36,37]. Therefore, based on its superior signal quality (SNR) and computation efficiency, PhysMamba [32] was selected as the optimal front-end extractor. For implementation, the selected model was applied to 128-frame chunks of the videos using the DiffNormalized scheme [38].

### 3.2. Overall Framework

**Figure 1:**
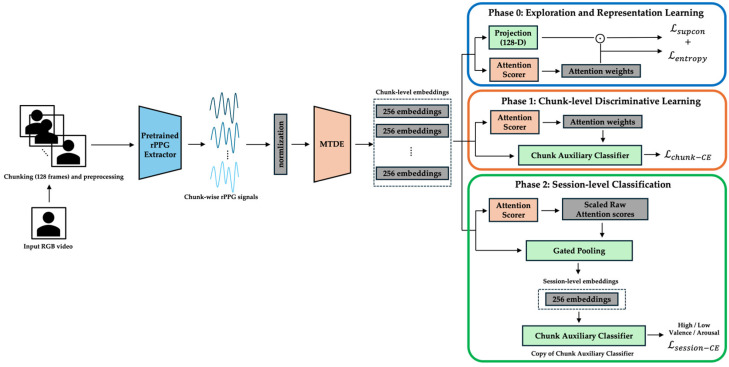
End-to-end pipeline for rPPG-based emotion recognition. An RGB video is separated into 128-frame segments (≈4 s), converted to rPPG waveforms using a pretrained PhysMamba, encoded using the Multi-scale Temporal Dynamics Encoder (MTDE), and optimized through Phase 0, Phase 1, and Phase 2, which are delimited by blue, orange, and green outlines, respectively. The arrows indicate the direction of the data flow and processing sequence.

**Figure 2:**
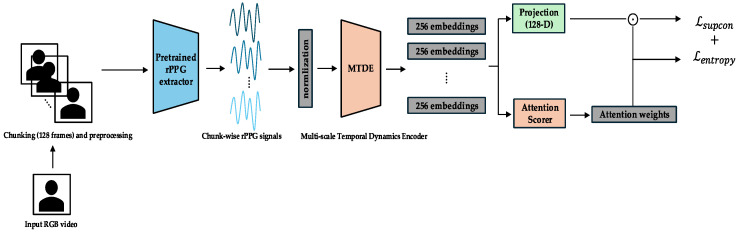
Phase 0—exploration and representation learning. MTDE embeddings are projected into a 128-dimensional space and trained with Supervised Contrastive Loss, while an internal SoftMax attention head is regularized by entropy; Gated Pooling and the session classifier remain frozen. The arrows indicate the direction of the data flow and processing sequence.

**Figure 3:**
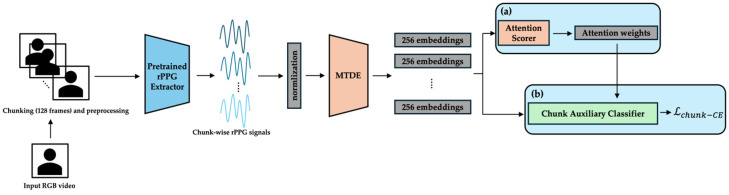
Phase 1—chunk-level discrimination and attentional refinement. (a) Top-K α-Entmax attention selects the most informative chunks; (b) the chunk auxiliary classifier is trained with focal cross-entropy, and Gated Pooling parameters are unfrozen from epoch 25. The arrows indicate the direction of the data flow and processing sequence.

**Figure 4:**
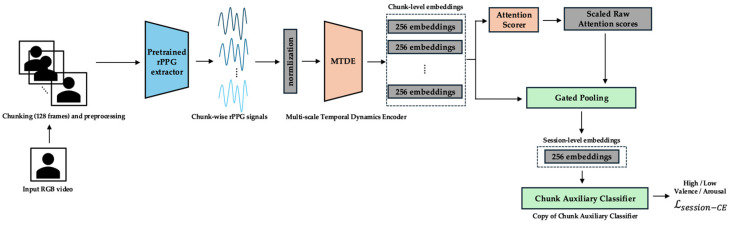
Phase 2—session-level inference and optimization. α-Entmax weights each chunk, Gated Pooling aggregates gated features into a 256-dimensional session vector, and the main classifier predicts high/low valence–arousal labels using session-level cross-entropy loss. The arrows indicate the direction of the data flow and processing sequence.

The complete end-to-end pipeline, together with its three curriculum phases, is shown in Figure 1. Raw RGB video is segmented into 128-frame chunks (≈4 s at 30 fps) and converted to rPPG waveforms by a pretrained PhysMamba extractor. Each chunk is then encoded by the Multi-scale Temporal Dynamics Encoder (MTDE). Depending on the training phase, different modules become active: the projection head and $L_{supcon}$ in Phase 0 (Figure 2), the chunk auxiliary classifier and Top-K α-Entmax attention in Phase 1 (Figure 3), and the Gated Pooling plus session classifier in Phase 2 (Figure 4). At inference time, the green-outlined path in Figure 1 (MTDE → α-Entmax → Gated Pooling → Main Classifier) constitutes the operational model.

Our emotion recognition model processes the 1D temporal rPPG signal, derived from the video, in fixed-length, non-overlapping temporal chunks of 128 frames (approximately 4 s at 30 fps). This specific chunk size was strategically chosen for multiple reasons. Firstly, it aligns with the temporal window used by the robust PhysMamba rPPG extractor [32] and common processing units in the rPPG-Toolbox framework [38]. Secondly, and crucially, prior work [23] has demonstrated that a 4-s segmentation size yields optimal performance for emotion classification from contactless PPG signals, reinforcing its appropriateness for capturing pertinent physiological dynamics within the temporal domain. Physiologically, a ≈4-s window is well-suited as it typically encompasses several cardiac cycles (e.g., approximately 4–7 heartbeats at a resting heart rate of 60–100 bpm). Analyzing physiological patterns such as heart rate variability (HRV) [5,15] or subtle pulse waveform changes, which are known to reflect autonomic nervous system dynamics [39] and have been effectively used for affective computing [6] over this duration, allows for the capture of meaningful short-term autonomic nervous system (ANS) modulations, which are widely recognized as crucial indicators of emotional states.

Each temporal chunk is processed using the Multi-scale Temporal Dynamics Encoder (MTDE), yielding a 256-dimensional feature embedding. The embeddings are scored by the attention scoring module (AttnScorer) via α-Entmax attention and, in parallel, forwarded to the Gated Pooling module (Gated Pooling). Gated Pooling integrates the attended and feature-gated embeddings into a single session representation from which the main classifier predicts the valence and arousal labels using only the aggregated temporal information. Phase-specific modules that augment this core path are illustrated in Figure 2 (Phase 0), Figure 3 (Phase 1), and Figure 4 (Phase 2) and are detailed in the curriculum learning section (Section 3.4).

### 3.3. Training Modules

This section provides a detailed description of the core modules constituting our emotion recognition framework.

#### 3.3.1. rPPG Extraction Front-End (PhysMamba)

As determined by our selection process (Section 3.1.3), the UBFC-rPPG-pretrained PhysMamba model [32,38]—chosen for its superior SNR and lower VRAM footprint—serves as the rPPG extraction front-end in our framework. Its primary role is to extract a refined, denoised 1D temporal rPPG (blood- volume pulse, BVP) signal from raw facial video frames.

PhysMamba operates on non-overlapping 128-frame chunks (≈4 s at 30 fps). Each chunk is first pre-processed with the DiffNormalized scheme [32,38], which computes frame-wise ratio differences normalized by the standard deviation, thereby enhancing subtle blood flow changes. The resulting 1D BVP waveform—produced without any additional per-session rescaling or band-pass filtering—constitutes the exclusive input to the downstream emotion recognition modules.

#### 3.3.2. Multi-Scale Temporal Dynamics Encoder (MTDE)

The MTDE’s purpose is to learn rich feature representations by capturing physiological dynamics across various temporal scales within each 128-frame BVP chunk. The design is physiologically grounded in the principle that the autonomic nervous system (ANS) modulates the cardiac pulse through multiple superimposed rhythms [40], with sympathetic and parasympathetic activities contributing to distinct frequency components of heart rate variability [15]. While these rhythms are classically studied via heart rate variability (HRV) frequency bands [41], our model does not compute HRV metrics explicitly. Instead, it learns features directly from the BVP waveform that are implicitly sensitive to the physiological processes underlying these HRV bands.

As detailed in Appendix A, the MTDE comprises two main stages: a SlimStem and a Multi-Scale Temporal Block (MSTB). The SlimStem consists of two sequential 1D convolutional layers for initial noise reduction and low-level feature extraction, conceptually akin to early sensory filtering. The subsequent MSTB implements our physiology-aligned strategy, featuring a three-branch architecture using dilated convolutions. Each branch is designed to have an effective receptive field (RF) that spans one of the key ANS timescales:

- Short-scale branch (RF ≈ 0.2 s): This scale is sensitive to rapid beat-to-beat changes in pulse morphology, reflecting high-frequency dynamics driven by parasympathetic (vagal) fluctuations, analogous to the HF-like band of HRV [4].
- Medium-scale branch (RF ≈ 2.2 s): This branch targets slower oscillations from the interplay between sympathetic and parasympathetic systems (e.g., baroreflex), operating in a window comparable to the LF-like band [4].
- Long-scale branch (RF ≈ 4.3 s): This branch integrates very slow modulations across the entire chunk, reflecting hormonal or thermoregulatory influences, which are thought to contribute to the VLF-like band [41]. Here, as the chunk is limited to 128 frames, the three RFs cover only the order-of-magnitude ranges rather than exact periods.

Outputs from the three MSTB branches are concatenated and passed through a SoftMax-based temporal attention pooling layer. This final step aggregates multi-scale information into a single, fixed-size 256-dimensional embedding ($h_{i}\;\in R^{D}$, where D=256) for each chunk.

#### 3.3.3. AttnScorer

The AttnScorer’s function is to evaluate each chunk embedding ($h_{i}$) and assign it a single scalar relevance score. This module is directly inspired by the physiological principle that autonomic responses to emotional stimuli are often transient and sparsely distributed in time [42]. Its purpose is therefore to learn to identify these brief, emotionally salient moments.

Architecturally, it consists of a 2-layer MLP with GELU activation (Appendix B). The raw attention scores are adaptively normalized using σ-γ scaling (a running-std gain control), a mechanism analogous to biological sensory normalization [43]. These scores are then transformed using α-Entmax attention [18,44], encouraging a sparse selection of salient chunks, which mirrors the selective focus of biological attention mechanisms [20].

#### 3.3.4. Gated Pooling

The Gated Pooling module aggregates the sequence of chunk embeddings ($h_{1},\;\ldots \;,\;h_{T}$) into a single, noise-resilient session-level representation ($h_{pooled}$). Its design is a direct response to the noisy nature of rPPG signals, where even within an emotionally salient chunk, not all feature dimensions are equally reliable [8].

It implements a learned, content-aware aggregation via two parallel mechanisms [45]. First, it uses the sparse attention scores ($a_{i}$) from the AttnScorer as temporal weights. Second, it computes a learned gate vector $g_{i}\;\in R^{D}$ that modulates each dimension via element-wise product ($g_{i}\;⨀\;h_{i}$), The final representation is computed as

(1) $$h_{pooled}\;=\;\sum_{i=1}^{T}\alpha_{i}\left(g_{i}\;⨀\;h_{i}\right)$$

This feature-level gating is pivotal, allowing the model to selectively amplify robust feature dimensions while suppressing those potentially corrupted by noise, a process inspired by neural gating and inhibition [46].

#### 3.3.5. Auxiliary Components

These modules are active only during specific curriculum phases (Section 3.4) to support learning objectives. The ChunkProjection module (Phase 0) is an MLP head for normalized embeddings used by the Supervised Contrastive Loss [47]. The ChunkAuxClassifier module (Phase 1) is a classifier attached before Gated Pooling module, predicting session labels from individual chunks to pretrain the MTDE for local discrimination. It is used to initialize the Main Classifier in Phase 2.

#### 3.3.6. Main Classifier

The Main Classifier receives the final aggregated session representation $h_{pooled}$ vector and predicts final emotion labels (low/high valence/arousal) via fully connected layers.

### 3.4. Training Curriculum

Our training employs a three-phase curriculum learning strategy, a methodology inspired by the developmental principle that meaningful learning occurs by progressively increasing the difficulty of training samples [21]. This structured approach is conceptually analogous to human skill acquisition, moving from broad feature perception to fine-grained discrimination and finally to integrated decision-making [22,48]. It systematically addresses the challenges of noisy, temporally sparse, and weakly labeled time-series data to achieve stable and effective learning. The specific pipeline configuration and module activations during each phase are illustrated in Figure 2, Figure 3 and Figure 4. Detailed hyperparameters for each phase are provided in Appendix C.

#### 3.4.1. Phase 0 (Epochs 0–14): Exploration and Representation Learning

- Cognitive/Computational Link: This initial phase corresponds to “pervasive exploration” observed in human learning, where a system first captures a wide array of sensory inputs to build a general understanding of the feature space before focusing on specific tasks. This aligns with classic models of attention where an initial, broad orientation precedes focused analysis [49].
- Objective: The primary objective is to train the MTDE and related components (AttnScorer and ChunkProjection) to produce robust and diverse embedding representations for individual temporal chunks. During this phase, the Gated Pooling module and the Main Classifier are not used for the primary loss computation.
- Primary Losses: The total loss in Phase 0 is a combination of the Supervised Contrastive Loss and an Entropy Regularization Loss. The Supervised Contrastive Loss ($L_{supcon}$) [47] is applied to the normalized embeddings from the ChunkProjection. This loss encourages embeddings from chunks originating from the same session (sharing the same label) to be closer in the representation space while pushing embeddings from different sessions apart.

The training in this phase is guided by a combination of two loss functions. The primary objective is the Supervised Contrastive Loss ($L_{supcon}$), defined as

(2) $$L_{supcon}\left(P\right)=\sum_{i\;\in \;I}\frac{-1}{\left|P\left(i\right)\right|}\sum_{p\in P\left(i\right)}log\left(\frac{exp\left(z_{i}{\;\cdot z}_{p}/\tau \right)}{\sum_{a\in A\left(i\right)}exp\left(z_{i}{\;\cdot z}_{a}/\tau \right)}\right)$$

Here, I is the set of anchor indices in the batch, $A\left(i\right)$ is the set of all indices in the batch except i, $P\left(i\right)$ is the set of indices of positive samples (same label) of i, z represents the normalized embedding vectors, and τ is the temperature parameter. To complement this, an Entropy Regularization Loss ($L_{entropy}$) is applied to the AttnScorer’s internal SoftMax attention output. Weighted by $\lambda_{entropy}$, this loss encourages a more uniform initial attention distribution, promoting a broader exploration of temporal features. This exploration is critical for preventing the model from prematurely converging on spurious patterns—a known risk related to the memorization capacity of deep networks, especially in the presence of noisy labels [50].

The overall loss for this phase combines both objectives.

(3) $$L_{Total}=\;L_{supcon}\;+\;\lambda_{entropy}L_{entropy}$$

#### 3.4.2. Phase 1 (Epochs 15–29): Chunk-Level Discrimination and Attentional Refinement

- Cognitive/Computational Link: This second phase corresponds to the establishment of selective attention. After the initial exploration, learning resources are focused on task-relevant signals. By using the AttnScorer and Focal Loss on only the Top-K chunks, our model progressively narrows its attentional focus, mimicking how neural systems learn to prioritize information-rich stimuli over time [51].
- Objective: The objective is to significantly enhance the discriminative capacity of the individual chunk embeddings and to refine the AttnScorer’s ability to identify emotionally salient temporal segments. During this phase, the ChunkProjection module is frozen, while the MTDE, AttnScorer, and ChunkAuxClassifier are actively trained.
- Primary Losses: The main objective is driven by a chunk-level classification task using the ChunkAuxClassifier. To handle class imbalance and focus on challenging examples, we employ Focal Loss [52] with γ=2.0 (Appendix C):

The primary loss function for Phase 1 is the chunk-level Focal Loss ($L_{Focal}=L_{chunk-CE}$), calculated as

(4) $$L_{chunk-CE}=L_{Focal}\left(p_{t}\right)=-\alpha_{t}{\left(1-p_{t}\right)}^{\gamma }log\left(p_{t}\right)$$

Here, we use a formulation where $p_{t}$ is the model’s estimated probability for the target class, $\alpha_{t}$ is a class-balancing weight, and γ is the focusing parameter.

Critically, this loss is calculated only for the Top-K chunks selected based on the AttnScorer’s α-Entmax output. The Top-K ratio, K, is strategically annealed downwards as training progresses. This forces the model to progressively focus its discriminative learning resources on the most salient segments, a process that mimics how human attention narrows onto key details within a stimulus [53].

Furthermore, this phase prepares the model for the next stage of training. The session-level cross-entropy loss ($L_{session-CE}$) is scheduled to be introduced from epoch 25, with its weight gradually ramping up. While the parameters for session-level components are unfrozen to allow for preparatory fine-tuning, $L_{session-CE}$ is not yet included in the total loss. This staged activation facilitates a smoother transition to Phase 2.

This staged activation strategy enables the model to begin adapting the session-level representation and pooling dynamics without prematurely influencing the optimization objective, thus facilitating a smoother transition to Phase 2 training. The overall loss for this phase is $L_{Total}=\;L_{chunk-CE}$.

#### 3.4.3. Phase 2 (Epochs ≥ 30): Session-Level Exploitation and Fine-Tuning

- Physiological/Cognitive Link: The final phase is analogous to evidence integration for decision-making [54]. The system leverages its refined representations and focused attention to weigh and combine the most reliable evidence distributed over time to form a final, consolidated judgment [55].
- Objective: The objective is to optimize the entire end-to-end pipeline for the final session-level emotion recognition task. The ChunkAuxClassifier is removed, and the Main Classifier is initialized with its weights. The MTDE, AttnScorer, Gated Pooling, and Main Classifier are all fine-tuned.
- Primary Loss Function: The sole objective function in this phase is applying the session-level cross-entropy loss ($L_{session-CE}$) to the output of the Main Classifier based on the Gated Pooling session embedding.

In this phase, the model is optimized using a session-level cross-entropy loss:

(5) $$L_{CE}\left(y,\;\hat{y}\right)=-\sum_{c=1}^{C}y_{c}log({\hat{y}}_{c})$$

Here, y is the one-hot encoded ground-truth label for the session, and $\hat{y}$ is the predicted probability distribution from the Main Classifier over C classes.

To achieve optimal session-level performance, key parameters are carefully scheduled to exploit the attentional patterns learned in the previous phase. Specifically, the AttnScorer is fine-tuned at a reduced learning rate (see schedule in Appendix C). Simultaneously, the $\alpha_{g}$ value for the α-Entmax transformation within the Gated Pooling module is annealed from 1.5 (at epoch 30) to 1.8 (at epoch 50). This increase in $\alpha_{g}$ promotes higher sparsity in the temporal attention weights, further refining the model’s focus onto the most crucial temporal segments for the final prediction.

### 3.5. Evaluation Metrics

Model performance was quantitatively evaluated using standard metrics on the independent test set. These metrics were chosen to provide a comprehensive and robust assessment, particularly considering potential class imbalances.

Performance was measured using accuracy and the weighted F1-score. The weighted F1-score is particularly valuable in the presence of class imbalances as it accounts for performance on all classes weighted by their frequency, providing a more objective measure than simple accuracy or macro-averaged metrics in such scenarios.

- Accuracy: This is defined as the proportion of correctly classified sessions out of the total number of sessions in the test set.

(6) $$Accuracy\;=\;\frac{Number\;of\;Correct\;Predictions}{Total\;Number\;of\;Predictions}$$

- Weighted F1-score: This metric is calculated based on the precision (${Precision}_{c}$), recall (${Recall}_{c}$), and F1-score (${F1}_{c}$) for each individual class c. The formulas for these class-specific metrics are

(7) $${Precision}_{c}\;=\;\frac{{TP}_{c}}{{TP}_{c}\;+\;{FP}_{c}}$$

(8) $${Recall}_{c}\;=\;\frac{{TP}_{c}}{{TP}_{c}\;+\;{FN}_{c}}$$

(9) $$F1_{c}\;=\;2\cdot \frac{{Precision}_{c}\;\cdot \;{\mathrm{Recall}}_{c}}{{\mathrm{Precision}}_{c}\;+\;{\mathrm{Recall}}_{c}}$$

The overall weighted F1-score is then computed as the average of the class F1-scores, weighted by the number of samples in each class ($N_{c}$):

(10) $$Weighted\;F1\;=\;\sum_{c=1}^{C}\frac{N_{c}}{N}\cdot F1_{c}$$

Here, N is the total number of samples in the test set, and C is the number of classes. In addition to these primary metrics, we also analyze the confusion matrix to gain insights into the model’s performance across different classes and the types of errors made.

### 3.6. Baseline

To contextualize the performance of our proposed framework, we selected the CNN-LSTM model by Mellouk & Handouzi [23] as our primary baseline. This choice was based on its status as the most domain-equivalent benchmark: it is a recent deep learning work that, similar to our study, uses contactless PPG for emotion recognition with approximately 4-s segments on the MAHNOB-HCI dataset.

However, a direct comparison faces significant limitation. Because Mellouk & Handouzi [23] did not make their source code available and do not disclose their specific test partition, it is impossible to run their model on our exact data split. Therefore, while we utilize their reported results for a comparative analysis, their performance should be interpreted as indicative rather than directly comparable. Our study mitigates this challenge by rigorously detailing our data split (Section 3.1) and employing the weighted F1-score (Section 3.5) to provide a more robust assessment that accounts for potential class imbalance. This approach establishes a clearer and more reproducible benchmark for future work.

Furthermore, to ensure a comprehensive evaluation and robustly validate our specific architectural choices, we implemented and compared our framework against a diverse set of alternative architectures. As detailed in Appendix E, these include a standard Temporal Convolutional Network (TCN), a 1D-CNN+LSTM hybrid model, and variants of our own model with different aggregation (Bi-LSTM) and attention (SE block) mechanisms. These extensive comparisons provide a clear justification for our design choices over various alternatives.

### 3.7. Experimental Setup

All experiments were conducted on a system with Ubuntu 20.04, Python 3.8, and PyTorch 2.1.2 (+CUDA 12.1), running on a single NVIDIA RTX 4080 GPU. A consistent batch size of 8 was used for all training phases of the emotion recognition model. Each item within a batch consisted of the complete sequence of temporal chunks belonging to a single session, ensuring that chunks from different sessions were never mixed within a sequence.

To manage GPU memory during the initial data preparation stage, long sessions were processed by the rPPG extractor in smaller segments (e.g., 24 chunks at a time). The resulting BVP waveforms were then concatenated to reconstruct the full session sequence before being used as input for model training.

For optimization, we employed the AdamW optimizer with a CosineAnnealingLR schedule. The learning rates were set to $3\times 10^{-4}$, $2\times 10^{-4}$, and $1\times 10^{-4}$ for Phases 0, 1, and 2, respectively, with weight decay set to $1\times 10^{-4}$ in Phase 0 and $5\times 10^{-4}$ for the subsequent phases.

In terms of computational resources, our proposed emotion recognition modules are lightweight. The front-end PhysMamba extractor consumes approximately 9.8 GiB of VRAM, while our entire pipeline has a maximum GPU memory consumption of 14.5 GiB under our batch configuration. This indicates that our downstream modules (MTDE, attention, etc.) add only about 4.7 GiB to the baseline load. The complete model has a total of 197,892 trainable parameters (164,996 for inference). On the aforementioned hardware, the average training time was approximately 4.1 min per epoch, and the average inference time for a 2-min session was 0.66 s, confirming the model’s efficiency and suitability for practical applications.

## 4. Results

### 4.1. Main Results

As summarized in Table 3 and Table 4, the proposed end-to-end framework demonstrates competitive performance in arousal classification using only the temporal rPPG signal from the MAHNOB-HCI dataset under a subject-mixed evaluation protocol. Specifically, the model achieves an accuracy of 66.04% and a weighted F1-score of 61.97%.

When compared against a conventional CNN-LSTM baseline [23]—evaluated using their reported confusion matrix—our model yields consistent improvements across all relevant metrics:

- Accuracy: 66.04% vs. 61.31%;
- Positive-class F1-score: 74.29% vs. 50.96%;
- Weighted F1-score: 61.97% vs. 59.46%.

These improvements validate the effectiveness of our architectural choices. Specifically, the MTDE is designed to be sensitive to the rapid waveform changes characteristic of sympathetic activation, while the sparse attentional chunk selection and feature-level Gated Pooling are adept at isolating these strong, transient physiological “events” from noisy segments. This event-driven design is particularly well-suited for capturing the discriminative temporal dynamics of arousal. The performance margin over the prior deep learning baseline affirms the merit of our tailored design for temporal-only physiological modeling. The corresponding confusion matrix of this result is provided in Appendix D (Table A5).

In contrast, and consistent with the known physiological limitations of this modality, valence classification remains a more complex and challenging task. Our model achieves a 62.26% accuracy and a 62.26% weighted F1-score, which are both substantially lower than those reported by the CNN-LSTM baseline (73.50% accuracy and 73.14% weighted F1-score calculated from their reported confusion matrix) [23]. The corresponding confusion matrix of this result is provided in Appendix D (Table A6).

This performance gap can be attributed to several fundamental factors:

- Physiological limitations: Arousal is closely associated with sympathetic nervous system (SNS) activity, which often manifests as acute, high-magnitude changes in cardiovascular patterns [56]. In contrast, valence is linked to more complex and subtle interactions involving the parasympathetic nervous system (PNS) and cortical patterns, which are not as easily captured in peripheral signals [4,57].
- Modality Constraints: By spatially averaging the facial region into a single 1D signal, our approach cannot access fine-grained spatial information. This is a notable limitation as recent studies suggest that regional facial blood flow patterns, such as subtle temperature changes around key facial areas, may hold valuable cues related to emotional valence [58].
- Architectural Inductive Bias: Our framework’s event-driven design, particularly its reliance on sparse attention to identify transient, high-magnitude physiological events, introduces an inductive bias. This approach is highly effective for detecting the “spikes” characteristic of SNS-driven arousal responses but is inherently less suited to integrating the subtle, sustained, and context-dependent patterns that often characterize valence.
- Data Imbalance: A notable class imbalance in valence labels may also contribute to biased predictions.

Collectively, these findings suggest that while our temporal rPPG modeling is potent for arousal recognition, effective valence modeling may necessitate either multimodal fusion or different architectural philosophies that prioritize long-term, contextual state integration over longer temporal windows.

### 4.2. Ablation Studies

To quantitatively assess the contributions of our key architectural components and the phased training strategy, we conducted a series of ablation studies. As our primary findings in Section 4.1 demonstrate that the proposed framework yields significant and meaningful improvements primarily in arousal classification, all subsequent ablation studies were centered on this task. This focused approach allows for a precise dissection of the components contributing to the model’s main success. Each study was designed to isolate the impact of a specific part of our framework on arousal recognition.

#### 4.2.1. Impact of Temporal Aggregation Strategy

First, to isolate the contribution of our pooling mechanism, we compared its performance against simpler aggregation strategies on the arousal task. All models in this test used the same MTDE backbone and were trained with the full curriculum. As shown in Table 5, replacing Gated Pooling with standard attention or average pooling resulted in a significant performance degradation of over 15% in accuracy. This finding underscores that a sophisticated, learned aggregation strategy is critical for effectively handling the noisy and complex temporal features extracted from rPPG signals.

#### 4.2.2. Validation of Multi-Scale Architecture in MTDE

To empirically validate our choice of a three-branch multi-scale architecture, we conducted an ablation study analyzing the impact of the number of parallel branches within the MTDE. We compared our proposed three-branch model against variants with one (a standard TCN), two, and four parallel branches. The detailed architecture of the TCN baseline is described in Appendix E. For the two-branch and four-branch variants, we used specific combinations of our predefined scales: the two-branch model combined our short- and long-scale branches, while the four-branch model added an additional medium-scale branch with a different dilation factor to test the impact of increased complexity.

The results presented in Table 6 reveal a clear trend that supports our three-branch design as the optimal configuration. The four-branch model showed the lowest performance among the multi-scale variants, suggesting that simply adding more branches leads to model over-parameterization and redundancy without providing additional discriminative information from the four-second chunks.

The TCN (single-branch) model, while outperforming some overly complex configurations, still fell short of our proposed model. This indicates that although it is a strong temporal model, a single receptive field is insufficient to capture the diverse range of physiological dynamics (from rapid morphological changes to slower oscillations) present in the BVP signal.

The two-branch model performed better than the single-branch TCN, confirming the benefit of a multi-scale approach. However, it did not surpass our proposed three-branch model, suggesting that incorporating the full spectrum of short-, medium-, and long-term dynamics is crucial for achieving the best performance.

These findings provide strong empirical support that our three-branch, physiologically aligned MTDE achieves the optimal trade-off between model complexity and the capacity to represent the multi-faceted temporal dynamics essential for emotion recognition.

#### 4.2.3. Ablation Study on Pooling and Attention Mechanisms (Arousal)

We also evaluated the impact of each core component within our three-phase curriculum learning strategy. As presented in Table 7, the full curriculum achieved the highest performance, and removing any key mechanism resulted in a clear degradation, confirming that each phase provides an essential contribution to the final model performance.

The most significant performance drop occurred with direct training (Phase 2 only), highlighting the fundamental need for a structured curriculum when dealing with noisy and weakly labeled data. This outcome empirically validates our core premise that a progressive, physiologically inspired learning strategy, analogous to human skill acquisition, is crucial for robust learning from complex physiological signals [21,22,48]. Removing the exploratory representation learning of Phase 0 (w/o SupCon) also significantly impaired the model’s ability to form a useful feature space, underscoring the importance of contrastive pre-training in such tasks. This observation aligns with the “pervasive exploration” principle in human learning, where an initial broad understanding of the feature space is foundational before focused analysis can occur [49].

Interestingly, the model trained without Top-K annealing yielded a slightly higher weighted F1-score despite its lower accuracy. A closer analysis revealed that this is not merely a statistical artifact of class imbalance but a direct consequence of the interplay between our curriculum strategy and the physiological nature of arousal. High arousal often manifests as strong, transient “events” in the BVP signal (e.g., sympathetic spikes), providing a clear and highly discriminative learning signal. Our Top-K annealing mechanism, designed to progressively focus on the most informative segments, naturally learns to prioritize these unambiguous high-arousal events. This transforms the model into a highly specialized “high-arousal detector,” achieving perfect recall for that class. This specialization is a direct computational manifestation of “selective attention,” where the model learns to prioritize information-rich stimuli akin to how neural systems focus on key details [51,53]. However, this specialization comes at the cost of a prediction bias as the model becomes more likely to misclassify the subtler, less event-like patterns of low-arousal states. The model without this refinement mechanism is less specialized, leading to a more balanced, albeit less accurate, performance across classes.

This finding provides a deep insight: our curriculum is highly effective at forcing the model to learn the most salient physiological features, but this very effectiveness can lead to a predictable bias when one class (high arousal) presents a much clearer, more “learnable” signal than the other.

### 4.3. Qualitative Analysis of Sparse Attention

The effectiveness and interpretability of our sparse attention mechanism are paramount, particularly its ability to prioritize physiologically meaningful regions [59]. We conducted a detailed visualization of the attention weights from our final model (with α-Entmax) and compared it against a standard attention model (with SoftMax) overlaid on a representative ECG signal from a random subject’s random session (Figure 5). This analysis provides direct insights into how our model’s attention aligns with critical physiological events.

As illustrated in Figure 5b, the standard Softmax attention produces a diffuse distribution across the temporal chunks, assigning moderate weights broadly without a clear, specific focus. This behavior suggests a lack of selective attention, where the model processes all chunks with similar emphasis, potentially diluting the significance of critical events. Quantitatively, this diffuse characteristic is reflected by its higher entropy value of 2.92. In stark contrast, Figure 5c clearly demonstrates that our α-Entmax attention learns a highly sparse distribution, evidenced by its significantly lower entropy value of 1.14, signifying a strong and concentrated allocation of attentional weight on a select few temporal chunks.

Crucially, Figure 5d distinctly shows that α-Entmax attention correctly identifies and assigns almost all of its attentional weight to specific temporal chunks that robustly coincide with significant physiological events in the ECG signal—specifically, clusters of high-frequency R-peaks. These R-peaks are widely recognized as key indicators of cardiac electrical activity and can signify sudden sympathetic arousal responses, making them physiologically meaningful moments [59,60]. This visual comparison provides a compelling demonstration of α-Entmax’s ability to filter out less relevant information and pinpoint critical temporal chunks, directly supporting its claim to mimic biological selective attention.

This visualization provides strong evidence that our sparse attention mechanism profoundly enhances model interpretability. By learning to selectively focus on physiologically meaningful moments through a carefully designed training curriculum, α-Entmax substantiates its interpretability and alignment with physiological principles, addressing critical considerations for robust scientific claims.

## 5. Discussion

Our proposed framework demonstrates promising capabilities in recognizing emotions exclusively from temporal remote photoplethysmography (rPPG) signals, achieving competitive arousal classification performance (66.04% accuracy; 61.97% weighted F1-score) and outperforming the CNN-LSTM baseline [23]. This superior arousal performance stems from our physiologically inspired temporal modeling and neuro-cognitively motivated learning strategy. The Multi-scale Temporal Dynamics Encoder (MTDE) effectively captures ANS modulations across timescales [4,15,39,40,41], showing that it is adept at detecting rapid pulse morphology changes indicative of sympathetic activation. The adaptive sparse α-Entmax attention [17] mirrors human selective attention [18,19,43,51,53], precisely identifying transient, salient physiological events like R-peak clusters (Figure 5) [59,60]. Robust aggregation via Gated Pooling [20,45], inspired by neural gating principles [46], further mitigates noise. Critically, our staged curriculum learning strategy [21], analogous to human skill acquisition [22,48,49], ensured stable and effective learning by systematically addressing data challenges [50]. Our ablation studies (Section 4.2) validate each component’s significant contribution to these gains.

Conversely, valence recognition remains significantly challenging with temporal rPPG signals. Our model achieved lower valence performance (62.26% accuracy; 62.26% weighted F1-score) compared to Mellouk & Handouzi’s baseline [23] (accuracy: 73.50%; weighted F1-score: 73.14%). This discrepancy highlights fundamental physiological limitations and inherent methodological constraints in valence detection using only temporal, spatially averaged rPPG data.

Physiologically, arousal is largely driven by sympathetic nervous system (SNS) activation [4,56], manifesting as acute, high-magnitude cardiovascular changes. These “event-like” shifts are well-captured by our framework’s design. In contrast, valence involves more intricate and subtle physiological responses, including a complex interplay between the sympathetic and parasympathetic nervous systems (PNS) [4,57] and higher-order cortical processing not directly reflected in peripheral cardiovascular changes. Positive valence often involves parasympathetic activation, while negative valence can manifest as nuanced patterns of co-activation or withdrawal, less pronounced than arousal’s overt responses.

Furthermore, subtle spatial variations in facial blood flow patterns [58] may carry valence-related cues, but our single, unidimensional temporal rPPG signal inherently loses this information. Cognitively, arousal often correlates with immediate, attention-grabbing physiological “events,” whereas valence requires integrating more subtle, sustained, and context-dependent patterns over longer temporal windows [42,54,55]. Our framework’s strong inductive bias towards transient, high-magnitude physiological events, effective for arousal, is thus less suited for these nuanced valence patterns. These findings strongly suggest that while temporal rPPG robustly reflects general ANS arousal, recognizing nuanced affective dimensions like valence likely necessitates multimodal integration (e.g., facial expressions and electrodermal activity) or advanced spatial–temporal rPPG analysis.

Regarding practical applicability, our approach demonstrates notable computational efficiency. Considering that it achieves an inference speed of approximately 0.66 s for a two-minute video segment on an NVIDIA RTX 4080 GPU, our model operates faster than real time. Its compact size (164,996 parameters) further facilitates deployment in resource-constrained scenarios (e.g., mobile devices and edge computing), significantly enhancing the viability of our temporal-only rPPG approach for unobtrusive affective computing where dedicated sensors or multiple modalities may not be feasible. This highlights the potential of specialized temporal models even within unimodal signal limitations.

### 5.1. Limitations

Our study, despite advancing temporal rPPG-based emotion recognition, presents several limitations informing future research. Firstly, our evaluation is confined to the MAHNOB-HCI dataset. This was a deliberate choice to rigorously investigate the capabilities and limits of temporal-only rPPG in a controlled environment with sufficient per-subject session data for deep learning. However, as reviewers noted, its limited scale (527 sessions) and diversity may constrain generalization to unseen subjects or broader contexts.

Secondly, our restriction to temporal-only rPPG, the scope of this foundational investigation, inherently excludes valuable spatial rPPG information or complementary affective cues from other modalities. As evidenced by lower valence performance (Section 5), this highlights the constraints of relying solely on a spatially averaged temporal pulse signal for nuanced affective dimensions.

A further limitation involves the explicit validation of our physiologically inspired mechanisms. While qualitative analysis (Section 4.3) and ablation studies (Section 4.2) demonstrated that our attention mechanisms prioritize physiologically meaningful regions and multi-scale encoding’s effectiveness, deeper statistical correlation analyses with established physiological markers (e.g., specific HRV components) were beyond this study’s initial scope, which focused on implicit learning from the BVP waveform. Our current model thus abstracts and emulates neuro-physiological principles, and further work is needed to fully mimic and quantitatively validate these complex biological processes within its learned representations.

### 5.2. Future Work

Addressing identified limitations, several clear avenues exist for future research building upon our temporal processing foundations.

Firstly, to enhance generalization and robustness, validating our framework on larger, more diverse datasets (e.g., DEAP and WESAD) is crucial. This includes exploring scenarios with limited per-subject data but broad subject ranges, assessing adaptability to new individuals even without extensive calibration. We aim to develop personalization strategies (e.g., few-shot subject calibration, meta-learning, and transfer learning) for robust performance across varied populations and conditions.

Secondly, to improve nuanced affective dimensions like valence, future work should expand beyond unimodal temporal rPPG. This includes integrating spatial–temporal rPPG analyses preserving regional blood flow patterns and multimodal fusion with complementary affective cues (e.g., facial expressions and electrodermal activity). Evaluating our model on alternative emotional elicitation methods, such as large-scale VR-based datasets, would further strengthen validation and assess domain generalization.

Finally, building upon our current work that abstracts and draws inspiration from neuro-physiological mechanisms, a critical future direction is to develop models that more deeply mimic and quantitatively validate these biological processes. This involves conducting deeper analyses into learned representations, potentially exploring their statistical correlation with specific, well-defined physiological markers beyond HR estimation. Such research would provide critical insights into which physiological dynamics and temporal patterns best indicate different emotional states, fostering more interpretable, scientifically grounded affective computing models that genuinely replicate biological information processing rather than merely using biological analogies.

Collectively, our results establish foundational benchmarks and methodological insights for future advancements in unimodal physiological emotion recognition, clearly defining both the capabilities and inherent limitations of relying solely on temporal-only rPPG signals. Our approach’s comprehensive physiological and cognitive grounding, combined with rigorous evaluation, ensures robust, interpretable, and applicable outcomes, advancing the state of the art in this challenging area.

## 6. Conclusions

We introduced a physiologically inspired deep learning framework for recognizing emotional states exclusively from temporal remote photoplethysmography (rPPG). Our approach systematically addresses critical limitations—temporal sparsity, signal noise, and weak labeling—through the Multi-scale Temporal Dynamics Encoder (MTDE), adaptive sparse attention, Gated Temporal Pooling, and a structured three-phase curriculum learning strategy. Empirical evaluation confirmed competitive performance in arousal classification (66.04% accuracy; 61.97% weighted F1-score), surpassing previous deep learning baselines. Conversely, lower performance in valence classification (62.26% accuracy) reveals fundamental physiological constraints in using solely temporal cardiovascular signals, clearly demarcating the capability boundaries of unimodal rPPG signals.

These results establish robust methodological benchmarks and highlight promising directions for future exploration: incorporating spatially resolved rPPG analysis or multimodal integration could significantly enhance nuanced emotional inference. This study provides critical foundational insights and clear guidelines to advance affective computing towards more accurate, reliable, and interpretable physiological emotion recognition.

## Figures and Tables

**Table 1:** Performance comparison of rPPG extraction methods on MAHNOB-HCI’s ECG.

Method Category	Model/Algorithm	Pre-Trained on	MAE	RMSE
Deep Learning	PhysFormer	UBFC-rPPG	5.37	7.32
	PhysFormer	PURE	7.01	9.72
	PhysMamba	UBFC-rPPG	5.38	7.43
	PhysMamba	PURE	6.16	8.75
Traditional	ICA	-	13.02	16.34
	POS	-	12.86	15.96
	CHROM	-	13.31	16.80
	GREEN	-	16.90	20.44
	LGI	-	15.76	19.28
	PBV	-	16.12	19.58
	OMIT	-	15.78	19.30

**Table 2:** Head-to-head performance comparison on UBFC-rPPG intra-dataset test.

Model	MAE	RMSE	MAPE	Pearson (ρ)	SNR (db)	VRAM (GiB)
PhysFormer	0.53	1.18	0.76	0.9994	0.25	10.1/16.3
PhysMamba	0.35	0.79	0.51	0.9997	3.67	9.8/16.3

**Table 3:** Main performance results of arousal classification (accuracy, F1-score for positive class, and weighted F1-score) on MAHNOB-HCI test set (53 unseen subjects) with subject-mixed split. Highlights performance solely from temporal domain. All metrics are reported as percentages.

Method	Accuracy (%)	F1 of Positive (%)	Weighted F1 (%)
CNN-LSTM [23]	61.31	50.96	59.46
Ours	66.04	74.29	61.97

**Table 4:** Main performance results of valence classification (accuracy, F1-score for positive class, and weighted F1-score) on MAHNOB-HCI test set (53 unseen subjects) with subject-mixed split. Highlights performance solely from temporal domain. All metrics are reported as percentages.

Method	Accuracy (%)	F1 of Positive (%)	Weighted F1 (%)
CNN-LSTM [23]	73.50	76.23	73.14
Ours	62.26	66.67	62.26

**Table 5:** Performance comparison across pooling strategies (arousal classification).

Method	Accuracy (%)	Weighted F1 (%)
Ours (MTDE + Gated Pooling)	66.04	61.97
MTDE + Attention Pooling	50.94	47.56
MTDE + Average Pooling	50.94	39.07

**Table 6:** Ablation study on the number of MTDE branches (arousal classification).

Feature Extractor Architecture	Accuracy (%)	Weighted F1 (%)
Ours (3-branch MTDE)	66.04	61.97
4-branch MTDE	54.72	54.72
2-branch MTDE	62.26	60.42
TCN (Single-branch)	58.49	52.05

**Table 7:** Ablation study on curriculum learning strategy (arousal classification).

Training Strategy	Accuracy (%)	Weighted F1 (%)
Full Curriculum (Phase 0→2)	66.04	61.97
Full Curriculum (w/o Top-K)	64.15	63.89
Phase 0 → 2 (w/o Auxiliary Classifier)	58.49	58.48
Phase 1 → 2 (w/o SupCon)	54.72	55.19
Phase 2 (Direct Training)	50.94	34.18

## Data Availability

The source code and trained weights are available at https://github.com/LeeChangmin0310/ReMOTION-Temporal (accessed on 7 May 2025). The MAHNOB-HCI dataset analyzed in this study can be obtained from the original authors under the CC-BY-NC-SA license. The repository includes the training scripts, inference demo, and the raw split lists used for reproducibility. Experimental environment details are also provided in the repository’s README, consistent with the setup described in Section 3.7.

## Abbreviations

The following abbreviations are used in this manuscript:

Acc	Accuracy
ANS	Autonomic Nervous System
AttnScorer	Attention Scorer
BVP	Blood Volume Pulse
CE	Confusion Matrix
CM	Convolutional Neural Network
EDA	Electrodermal Activity
HRV	Heart Rate Variability
MIL	Multiple Instance Learning
MTDE	Multi-scale Temporal Dynamics Encoder
rPPG	Remote Photoplethysmography
WF1	Weighted F1-Score

## Appendix A. Architecture Details of MTDE

### Appendix A.1. Architecture Overview

The MTDE encodes each 128-frame (4 s) rPPG chunk into a 256-dimensional embedding through two stages:

- SlimStem: Two Conv1D layers (kernel = 5, then 3 with stride = 2) for initial noise reduction and temporal downsampling (T = 128 → 64).
- MultiScaleTemporalBlock (MSTB): Three parallel branches with different kernel sizes and dilations are used to model short-, mid-, and long-range temporal dynamics.

### Appendix A.2. Physiological Rationale and Receptive Fields

**Table A1:** MSTB’s parameters per branch.

Branch	Kernel	Dilation	Effective RF	Approx. Duration	Physiological Role
Short	3	3	6	~0.2 s	Parasympathetic (vagal) fluctuations, analogous to HF band of HRV.
Medium	5	8	66	~2.2 s	Sympathetic–parasympathetic interplay (e.g., baroreflex), analogous to LF band.
Long	3	32	129	~4.3 s	Very slow modulations (e.g., hormonal and thermoregulatory), analogous to VLF band.

Here, the receptive field can be calculated by $RF=\left(k-1\right)\times d+1$.

### Appendix A.3. Pooling Layer: SoftMax Pool

SoftMax over time across temporal steps:

(A1) $$w=softmax\left(W_{attn}\cdot x\right),\;\;h\;=\;\sum_{t}w_{t}x_{t}$$

This results in a single (B, D) embedding per chunk. No gating is applied in this layer.

## Appendix B. Attention Modules: AttnScorer and Gated Pooling

### Appendix B.1. AttnScorer: Phase-Aware Attention Scoring

#### Appendix B.1.1. Architecture

- Two-layer MLP: Linear (D, D/2) → GELU → Linear (D/2, 1);
- Zero-mean score normalization: $s_{i}\;\leftarrow \;s_{i}-mean(s)$;
- σ−γ scaling with EMA-based adjustment:

(A2)
$$\gamma \;=\;\frac{\sigma_{target}}{\sigma_{run}+\varepsilon }\;\in \;[0.5,\;2.0]$$

- Raw score scaling: $s_{i}^{scaled}=\gamma \cdot s_{i}$.

Here, $\sigma_{run}$ is the running standard deviation of the raw attention scores, calculated via an exponential moving average. $\sigma_{target}$ is a scheduled hyperparameter representing the target standard deviation (e.g., 0.8 for early epochs and 1.2 for later epochs). The value of γ is clamped to the range [0.5, 2.0].

#### Appendix B.1.2. Phase-Dependent Attention Mechanism

**Table A2:** Attention type per phase.

Phase	Epoch Range	Attention Type	Notes
0	0–14	Softmax	Entourage diversity
1	15–29	α-Entmax ()	Sharp, sparse, differentiable Top-K
2	≥30	Raw scores only	Passed to Gated Pooling

#### Appendix B.1.3. Entmax Scheduling During Phase 1

(A3)
$$\alpha \left(e\right)=\left\{\begin{array}{ll} 1.0+0.3\;\cdot \frac{e-15}{5}, & if\;15\;\leq \;e<20\\ 1.3+0.3\;\cdot \frac{e-20}{5}, & if\;20\;\leq \;e<25\\ 1.7 & ,if\;25\;\leq \;e<30 \end{array}\right.$$

### Appendix B.2. Gated Pooling: Sparse Temporal Aggregation

Receives chunk embeddings $h_{i}\;\in R^{D}$ and raw attention scores. In Phase 2, the attention scores are transformed using $\alpha_{g}$-Entmax to produce temporal weights:

(A4) $$\alpha_{i}={entmax}_{\alpha_{g}}\left({raw\_score}_{i}\right),g_{i}\;=\sigma \left(MLP\left(h_{i}\right)\right)$$

The session-level pooled embedding is computed as

(A5) $$h_{pooled}\;=\;\sum_{i=1}^{T}\alpha_{i}\left(g_{i}\;⨀\;h_{i}\right)$$

where ⨀ denotes element-wise multiplication.

$\alpha_{g}$ is scheduled during Phase 2 as

(A6) $$\alpha_{g}(e)=1.7+0.3\;\cdot min\left(\frac{e-30}{19},\;1.0\right),\;where\;\;e>30$$

This dual mechanism (Temporal weight × Feature gate) reflects neural inhibition, enabling selective suppression of irrelevant dimensions even within salient chunks.

## Appendix C. Phase-Wise Training Schedule

**Table A3:** Epoch-based curriculum strategy.

Phase	Epochs	Objective	Active Modules
0	0–14	Embedding diversity (SupCon)	MTDE, AttnScorer, ChunkProjection
1	15–29	Chunk-level discrimination	MTDE, AttnScorer, ChunkAuxClassifier, Gated Pooling (E ≥ 25)
2	30–49	Session-level classification	Gated Pooling, Classifier

**Table A4:** Hyperparameter scheduling.

Epoch	Top-K Ratio	SupCon λ	CE λ	$\lambda_{entropy}$	τ (Temp)	α (AttnScorer)	$\alpha_{g}$ (Gated)
0	0.0	1.00	0.0	0.1	1.2	—	—
14	0.0	0.44	0.0	0.1	0.7	—	—
15	0.6	0.0	0.5	0.0	1.0	1.0	—
25	0.3	0.0	0.5	0.0	1.0	1.7	start = 1.7
30	—	0.0	0.7	0.0	1.0	raw only	1.7 → 2.0
50	—	0.0	1.0	0.0	1.0	raw only	2.0

## Appendix D. Confusion Matrices

This appendix provides the confusion matrices for arousal and valence classification results from the final proposed model evaluated on the MAHNOB-HCI test set.

**Table A5:** Confusion matrix—arousal classification (final model).

	Predicted Low	Predicted High
Actual Low	9	18
Actual High	0	26

- Accuracy: 66.04%;
- Weighted F1-score: 61.97%;
- The model shows strong sensitivity to high arousal states (recall: 100%), with most misclassifications occurring in the low-arousal category.

**Table A6:** Confusion matrix—valence classification (final model).

	Predicted Low	Predicted High
Actual Low	13	10
Actual High	10	20

- Accuracy: 62.26%;
- Weighted F1-score: 62.26%;
- The model demonstrates relatively balanced performance but reveals confusion between low and high valence categories, indicating the nuanced nature of valence detection from unimodal temporal signals.

## Appendix E. Confusion Matrices

To provide a comprehensive evaluation and validate our architectural choices, we compared our proposed framework against several strong alternative architectures. Each alternative was designed to test a specific component of our model, such as the feature extractor, temporal aggregator, or attention mechanism. For a fair comparison, all models were trained using the same three-phase curriculum on the arousal classification task.

### Appendix E.1. Description of Comparative Models

- Temporal Convolutional Network (TCN): A standard TCN with a stack of dilated causal convolutions was implemented to serve as a strong, non-recurrent backbone baseline. The TCN consisted of two residual blocks, each with two layers of dilated causal convolutions (kernel size = 7, 256 channels), and dilation factors following exponential growth (1, 2, 4, 8).
- 1D-CNN + LSTM: This is a classic hybrid architecture combining a simple 1D-CNN feature extractor with an LSTM layer to model sequential dependencies.
- Multi-scale + Bi-LSTM: This model replaces the final attention pooling layer of our MTDE with a Bi-directional LSTM to evaluate a standard recurrent aggregation method.
- Multi-scale + SE block: This model integrates Squeeze-and-Excitation (SE) blocks into our MTDE to assess the effect of a channel-wise attention mechanism as an alternative to our temporal attention.

### Appendix E.2. Result and Analysis

The results of the comparative evaluation are summarized in Table A7.

**Table A7:** Performance comparison with alternative backbones (arousal classification).

Comparison Focus	Backbone/Variant	Accuracy (%)	Weighted F1 (%)
Ours (3-branch MTDE)	Ours (3-branch MTDE)	66.04	61.97
Backbone Comparison	Standard TCN	58.49	52.05
	1D-CNN + LSTM	58.49	52.69
Aggregation Comparison	Multi-scale + Bi-LSTM	52.83	46.34
Attention Comparison	Multi-scale + SE block	60.38	52.50

The results consistently demonstrate that our proposed MTDE-based architecture outperforms all tested alternatives. A detailed analysis for each comparison is presented as follows:

- Backbone Analysis (MTDE vs. TCN, 1D-CNN+LSTM): The proposed MTDE outperforms both the standard TCN and the classic hybrid model. This suggests that for this specific task, the physiologically aligned receptive fields of the MTDE are more effective at capturing the relevant, multi-scale dynamics of the BVP waveform than a generic temporal model like TCN. The relatively lower performance of the LSTM-based variant may be attributed to the nature of our input data. Recurrent models are designed for long-range dependencies, but in our framework, which processes short, 4-s chunks, the most critical emotional cues are embedded in the local waveform morphology. In such cases, the added complexity of a recurrent layer may not provide a significant advantage.
- Aggregation and Attention Analysis (vs. Bi-LSTM, SE block): Our model also outperforms the variants with a Bi-LSTM aggregator or an SE block. This suggests that our combined temporal attention (AttnScorer) and feature-gating (Gated Pooling) mechanism is more effective than a standard recurrent aggregator. The underperformance of the SE block variant further implies that for this task, identifying when an emotionally salient event occurs (temporal attention) is more critical than re-weighting which feature channels are active (channel-wise attention).
- Note on Transformer-based Models: While Transformer architectures are powerful, we deliberately did not include them as a primary baseline for two reasons. First, Transformers are notoriously data-hungry and are prone to overfitting on datasets of limited scale and session diversity like MAHNOB-HCI. Second, their core strength lies in modeling very long-range global dependencies, which, as observed with the LSTM variants, may be suboptimal for our event-driven, short-chunk analysis. As our ablation studies suggest, for this specific problem context, increasing architectural complexity does not necessarily guarantee better performance.

Overall, these extensive comparisons validate that our tailored, physiology-aligned, and lightweight architecture provides the most effective solution for temporal rPPG-based emotion recognition under the given data constraints.
